# Identification of the Main Metabolites of a Marine-Derived Strain of *Penicillium brevicompactum* Using LC and GC MS Techniques

**DOI:** 10.3390/metabo10020055

**Published:** 2020-01-30

**Authors:** Francesco Vinale, Maria Michela Salvatore, Rosario Nicoletti, Alessia Staropoli, Gelsomina Manganiello, Tommaso Venneri, Francesca Borrelli, Marina DellaGreca, Francesco Salvatore, Anna Andolfi

**Affiliations:** 1 Department of Veterinary Medicine and Animal Productions, University of Naples Federico II, 80137 Naples, Italy; frvinale@unina.it; 2 Institute for Sustainable Plant Protection, National Research Council, 80055 Portici (NA), Italy; alessia.staropoli@unina.it; 3 Department of Chemical Sciences, University of Naples Federico II, 80126 Naples, Italy; mariamichela.salvatore@unina.it (M.M.S.); dellagre@unina.it (M.D.); frsalvat@unina.it (F.S.); 4 Council for Agricultural Research and Economics, Research Centre for Olive, Citrus and Tree Fruit, 81100 Caserta, Italy; rosario.nicoletti@crea.gov.it; 5 Department of Agricultural Sciences, University of Naples Federico II, 80055 Portici (NA), Italy; gelsomina.manganiello@unina.it; 6 Department of Pharmacy, School of Medicine and Surgery, University of Naples Federico II, 80131 Naples, Italy; tommaso.venneri@unina.it (T.V.); franborr@unina.it (F.B.)

**Keywords:** epidithiodioxopiperazines, marine-derived fungi, antiproliferative activity, *Anemonia sulcata*, mycophenolic acid, thiosilvatins, beneficial microbes

## Abstract

Marine-derived fungi are an important source of many valuable compounds with original structures and diverse physico-chemical properties. In this work, the metabolomic profile of a strain of *Penicillium brevicompactum*, recovered from a snakelocks sea anemone (*Anemonia sulcata*), was investigated through the parallel application of LC-ESI-HRMS, GC-MS, and NMR. Our strategy allowed the identification of mycophenolic acid, brevianamide A, and several compounds belonging to the thiosilvatins. Among the latter, five products are reported for the first time in this species. The main product of this series, *cis*-bis(methylthio)silvatin, was also tested for antiproliferative activity on both cancer and non-tumoral colon cell lines.

## 1. Introduction

Marine-derived fungi are unanimously considered a source of untapped chemodiversity to be exploited in view of drug discovery [1,2]. Within the many Ascomycetes reported from the sea environment, the genus *Penicillium* is particularly widespread in association with any kind of marine organism, and represents one of the most prolific taxa with reference to the production of bioactive compounds [3,4,5,6].

This paper reports the results of a metabolomics analysis of a strain of *Penicillium brevicompactum* (AN4) recovered from a snakelocks sea anemone (*Anemonia sulcata*), within an ongoing investigational activity on marine-derived fungi from Naples Bay (Italy) [7,8]. Novel data concerning the biological activity of one of the main products of this strain, *cis*-bis(methylthio)silvatin, are also reported.

## 2. Results

### 2.1. Profiling and Structural Elucidation of Secondary Metabolites from Penicillium brevicompactum AN4

The crude EtOAc extract obtained from culture filtrates of strain AN4 was analysed by LC-MS (Figure S1) and GC-MS and successively fractionated by combined column (CC) and thin layer chromatography (TLC), leading to the purification of two known compounds—mycophenolic acid and *cis*-bis(methylthio)silvatin—whose structures were confirmed by a comparison with data reported in the literature [9,10]. NMR spectra were reported in Figures S2–S6.

Both the crude EtOAc extract and its fractions were subjected to GC-EI-MS analysis, which was carried out following derivatization with the silylating reagent *N*,*O*-bis (trimethylsilyl) trifluoroacetamide (BSTFA). This derivatization procedure allows the sensitivity towards metabolites containing alcohol, phenol, carboxylic acid, and amine functional groups in their structure to be enhanced; in fact, these functions are readily converted to the TMS functional group (-Si(CH_3_)_3_) through substitution of the H atom of -OH and -NH functions [7].

The acquired gas chromatograms (Total Ion Current Chromatograms, TICC) showed that the EtOAc extract was very rich in metabolites. However, a number of products associated with well-developed chromatographic peaks in TICC could not be readily identified by analysing the spectrum in comparison with the custom MS libraries available in our laboratory, theNIST17 library, or other online databases. In particular, TICC showed nine main chromatographic peaks with a good purity which were associated with target metabolites in this investigation.

At this stage, the nine target metabolites were identified by their Kovats non-isothermal retention index (RI) and associated electron impact (EI) mass spectrum (Figure S7). The Kovats non-isothermal or, depending on the type of elution performed, isothermal retention index, is a way to express the retention of an analyte relative to the retention of n-alkanes (which are assumed as reference compounds on the Kovats retention index scale). The conversion of absolute retention times, which are erratic and strongly dependent on the chromatographic method settings, is convenient because it produces a number (the retention index) which is practically independent of the chromatographic conditions, so much so that it can be used to tag an unknown compound much in the same way as a compound can be tagged with its boiling point or other invariant property ([11] and literature therein). However, the conversion of retention times to corresponding retention indexes requires a standard mixture of n-alkanes to be chromatographed, with the same settings and instrument within a short time from acquisition of the analytical data.

Apart from that, although most of the separated fractions contained mixtures of compounds, two fractions each contained a single substance with a good degree of purity. These two fractions were subjected to NMR structural analysis, which led to the identification of *cis*-bis(methylthio)silvatin (C_20_H_28_N_2_O_3_S_2_) and mycophenolic acid (C_17_H_20_O_6_).

In order to identify the remaining seven targeted metabolites, chromatographic fractions containing one or more of these products were subjected to LC-ESI-HRMS analysis. To each of the non-identified target metabolites, an accurate value of the monoisotopic mass, within an uncertainty of ±0.0002–0.0005 u, was assigned from the high-resolution LC-ESI-HRMS mass spectra (Figures S8–S15). From the accurate mass, the atomic composition of each target metabolite was readily derived by using standard MS tools.

**Figure 1:**
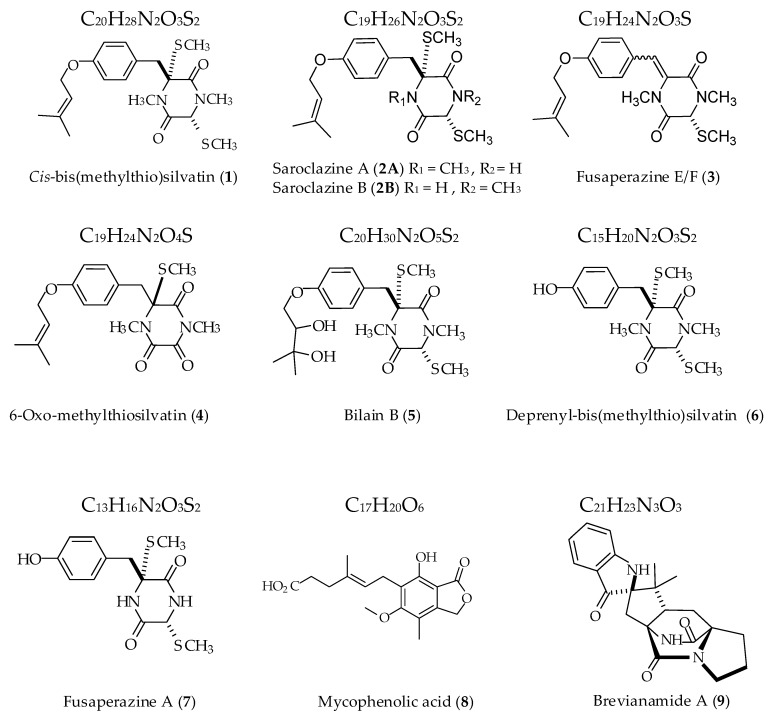
Structures of target secondary metabolites (**1**–**9**) identified in the organic extract of strain AN4.

The accurate mass and formulas associated with each product are reported in Table 1. The data in Table 1, and the corresponding EI mass spectra, were employed to assign a structure to each of the unidentified target metabolites. Hence, LC-ESI-HRMS and GC-EI-MS allowed the putative identification of other compounds (Figure 1), a number of which were found to belong to the thiosilvatin series [12].

It is to be specified that, unlike *cis*-bis(methylthio)silvatin (**1**), whose configuration was assigned by NMR, the determination of stereochemistry was not possible for compounds identified via MS. Therefore, their configuration in Figure 1 was arbitrarily assigned.

### 2.2. Bioassay

**Figure 2:**
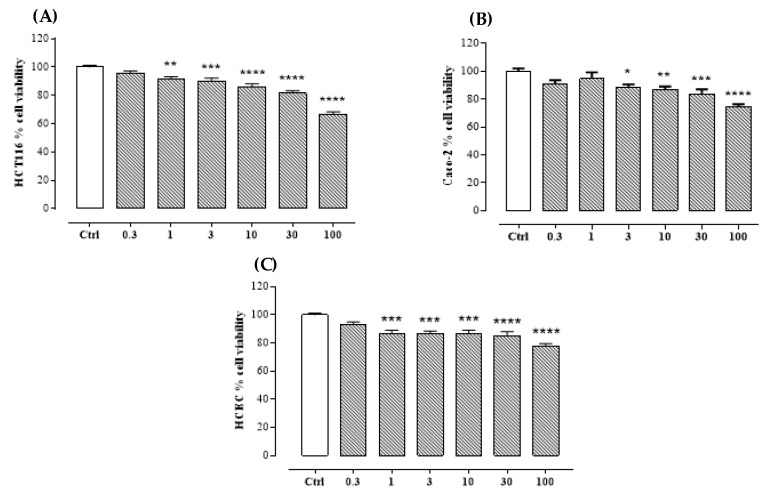
Effect of cis-bis(methylthio)silvatin (0.3–100 µM) on cell viability, evaluated by the MTT assay, in the colorectal cancer cell lines HCT116 (**A**) and Caco-2 (**B**), and in the non-tumoral colonic epithelial cell line HCEC (**C**). Cells were incubated with an increasing concentration of the compound (24 h exposure). Each bar represents the mean ± SEM of three independent experiments. * *p* < 0.05, ** *p* < 0.01, *** *p* < 0.001, and **** *p* < 0.0001 versus control (ctrl, untreated cells).

The cytotoxic activity of cis-bis(methylthio)silvatin was evaluated at different concentrations (0.3–100 µM) on two colorectal cancer cell lines (HCT116 and Caco-2) and a non-tumoral colonic epithelial cell line (HCEC). By using the MTT assay, we found that cis-bis(methylthio)silvatin significantly reduced the cell viability in HCT116 and Caco-2 cells in a concentration-dependent manner, starting from 1 µM for HCT116 and 3 µM for Caco-2 cells (Figure 2A,B), with EC_50_ (i.e., the concentration giving the half-maximal response) values of 29.29 and 35.31 µM, respectively. Similarly, the compound exerted cytotoxic activity on HCEC, with a significant effect starting from 1 µM and an EC_50_ value of 0.57 µM (Figure 2C).

## 3. Discussion

Fungal metabolites have very diverse physico-chemical properties and occur at different abundance levels. Consequently, a comprehensive metabolomics investigation is a challenge for researchers working on natural product discovery, in view of performing a complete screening of detectable compounds in samples obtained from culturing.

To perform an accurate identification of secondary metabolites produced by strain AN4, the parallel application of several instrumental techniques was employed (i.e., NMR, LC-ESI-HRMS, and GC-MS).

An important question to be discussed concerns the way the structures presented in Figure 1 have been derived from the data in Table 1 and the associated EI mass spectra collected via a GC-EI-MS analysis of fractions from the EtOAc extract of strain AN4.

**Figure 3:**
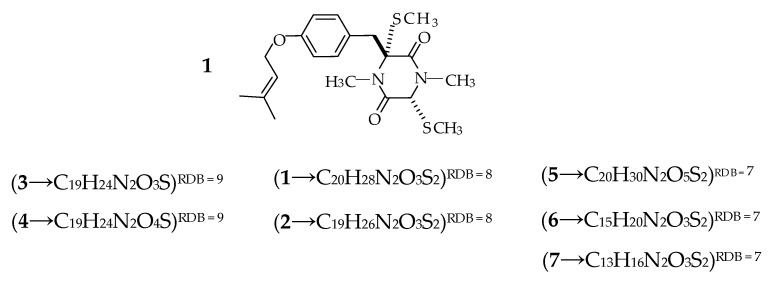
Comparison of *cis-*bis(methylthio)silvatin (**1**) and metabolites **2**–**7** for structural assignment.

By considering the formula and number of ring and double bonds (RDB) of metabolites **1**–**7** in Table 1, as presented in Figure 3, one can hardly avoid the perception that they refer to a group of structurally related molecules, probably being part of a common metabolic pathway.

As it can be deduced from Figure 3, the RDB = 8 number of (**1**), whose structure was unambigously assigned by NMR analysis of the purified compound, is accounted for: four RDB of the benzene ring, one RDB of the heterocyclic 6-membered ring, two RDB of two C = O functional groups, and one RDB of the double bond in the alkyl side chain.

The RDB of the targeted metabolites **2**–**7** ranges between 7 and 9, so all of them can accommodate the benzene ring and several additional rings and/or double bonds. Furthermore, all the compounds in Figure 3 present two nitrogen atoms which are involved in the formation of the six-membered heterocyclic ring.

Based on these considerations, it is a reasonable starting point to assume that the structures of **2**–**7** share the same basic structural pattern of **1**. This fundamental consideration can be used in various ways to tentatively assign a structure to our targeted metabolites.

One of the most obvious approaches is to make a similarity structure search in a database of known chemical molecules (e.g., PubChem), starting with the known structure of **1**.

A second, very intriguing approach is to exercise, guided by the pertinent EI mass spectra, our chemical understanding and knowledge on breaking and closing bonds, to translate the differences between formulas of unidentified metabolites and compound **1** into structural variations**.** This function can also be supported by an ad hoc program which is capable of predicting mass spectra from a hypothesized structure on the basis of well-established fragmentation rules. For this purpose, we used the NIST program MS Interpreter [13], which is a well-known MS fragmentor and which can be freely downloaded from the internet.

As an example of the procedure we have employed throughout to assign a structure to metabolites from **2** to **7** (and which is sketched for each single metabolite in the supplementary material), we discuss, in the following, as briefly as possible, how a structure can be associated with the target metabolite **2** on the basis of data in Table 1, its EI mass spectrum, and the EI mass spectrum of the fully characterized *cis*-bis(methylthio)silvatin (**1**).

First, if we compare the formula of **2** (C_19_H_26_N_2_O_3_S_2_) with the formula of **1** (C_20_H_28_N_2_O_3_S_2_), we immediately see that these two molecules, which have the same RDB number, simply differ by a CH_2_ group. When we look at the structure of **1** in Figure 3, we see that there are several ways in which metabolite **2** can be generated by the substitution of a -CH_3_ group with hydrogen.

**Figure 4:**
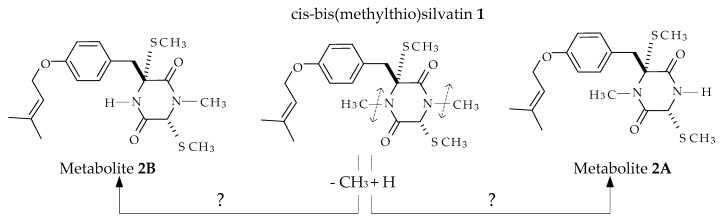
Hypothetical pathways for the conversion of the formula of *cis*-bis(methylthio)silvatin (**1**) to the formula of metabolite **2** in Table 1.

For better clarification, two possible conversion processes, which produce supposed structures for metabolite **2**, are described in Figure 4. Please note that although structures **2**A and **2**B, hypothesized in Figure 4, refer to two different isomeric compounds, in the following, we will treat them as a single hypothetical structure for metabolite **2** (since it can be anticipated that it will be impossible to distinguish between them on the basis of MS data).

**Figure 5:**
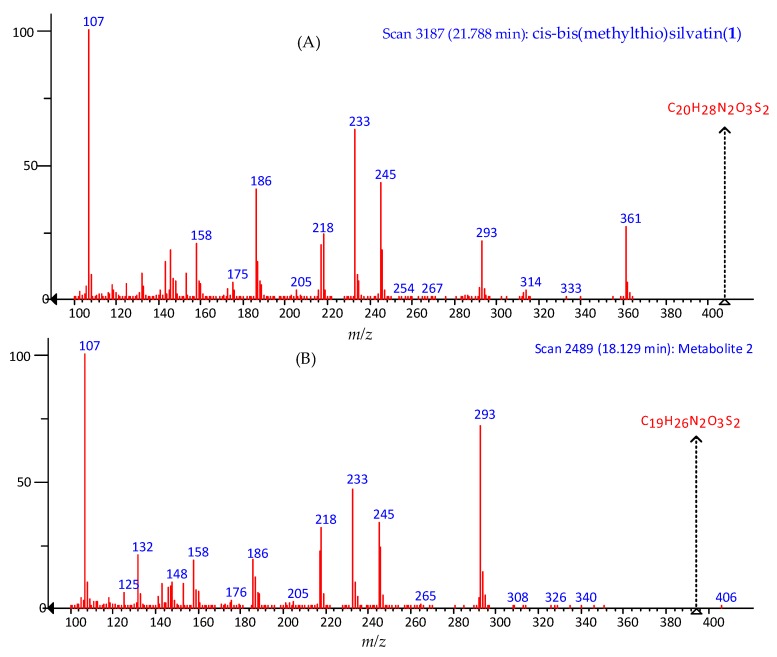
Comparison of 70 eV electron impact (EI) MS of the NMR-characterized *cis*-bis(methylthio)silvatin (**1**), (**A**), with the spectrum of the unidentified target metabolite 2, (**B**).

Second, we can provide substantial support for the hypothetical structure in Figure 4, by confronting the EI mass spectra of *cis*-bis(methylthio)silvatin (**1**) with the mass spectrum of metabolite **2**, as is shown in Figure 5.

As can be seen, the EI mass spectra of both compounds do not exhibit a molecular ion mass peak. However, this is irrelevant in the present case because we already know the empirical formula of the two compounds (which has been derived from HR MS data, see Table 1).

Even a superficial inspection of Figure 5 will show a strict correlation of the mass spectra, which obviously derives from structural relationships. For instance, well developed mass peaks are present at *m*/*z* 107, 158, 186, 233, 245, 293, etc., in both spectra. The main difference between the two spectra appears to be a peak at *m*/*z* 361, which is only present in the spectrum of *cis*-bis(methylthio)silvatin (**1**).

**Scheme 1:**
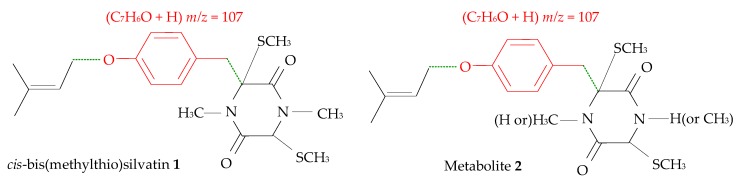
Substructure colored in red has been assigned to ion C_7_H_7_O^+^ at *m*/*z* = 107 in Figure 5, while substructures in black represent the neutral losses for its formation from the parent ion.

In primis, we consider that the base peak, both in spectrum (A) and (B), appears at *m*/*z* = 107. This peak is readily assigned to the ion C_7_H_7_O^+^, whose composition is derived from the analysis of its well-developed isotopic pattern. In addition, the substructure colored in red in Scheme 1 is assigned to this fragment on the basis of the known structure of *cis*-bis(methylthio)silvatin (**1**).

This assignment is also supported by MS Interpreter, which readily connects the red colored substructure to the *m*/*z* 87 peak in the mass spectrum of *cis*-bis(methylthio)silvatin (**1**).

From the above, there can be no doubt that cis-bis(methylthio)silvatin (**1**) and metabolite **2** share the substructure drawn in red in Scheme 1.

**Scheme 2:**
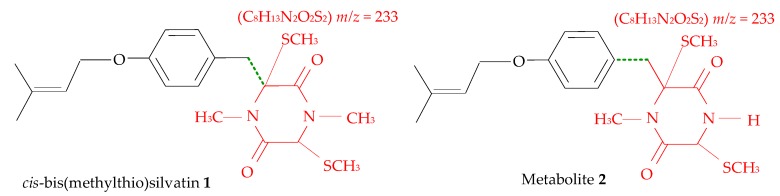
Substructure colored in red has been assigned to ion C_8_H_13_N_2_O_2_S_2_^+^ at *m*/*z* = 233 in Figure 5, while the substructure in black represents the neutral loss for its formation from the parent ion.

The second most abundant mass peak in the spectrum of *cis*-bis(methylthio)silvatin (**1**) displays *m*/*z* 233. The formula C_8_H_13_N_2_O_2_S_2_^+^ is assigned to this peak from its isotopic pattern. In Scheme 2, it is shown that a fragment of composition C_8_H_13_N_2_O_2_S_2_^+^ can be readily created from the structure of *cis*-bis(methylthio)silvatin (**1**) by simple scission of the benzylic σ bond (benzylic cleavage).

However, the analogous benzylic scission in the hypothetical structure of metabolite **2** would create the fragment C_7_H_11_N_2_O_2_S_2_^+^, which would appear as a peak at *m*/*z* 219, and this is not the case.

In Scheme 2, it is shown how this apparent contradiction can be resolved by simply replacing scission at the benzylic position (which takes place for *cis*-bis(methylthio)silvatin (**1**)) with scission of the phenylic σ bond (which presumably predominates for metabolite **2**).

The mass peak at *m*/*z* 293 is also shared by *cis-*bis(methylthio)silvatin (**1**) and metabolite **2**.

**Scheme 3:**
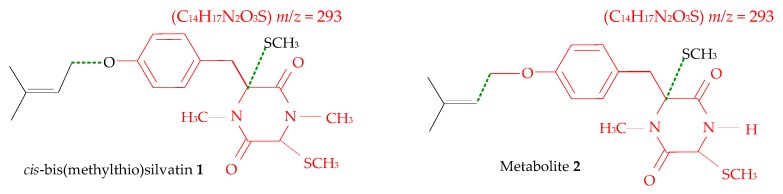
Substructure colored in red has been assigned to ion C_14_H_17_N_2_O_3_S^+^ at *m*/*z* = 293 in Figure 5, while substructures in black represent the neutral losses for its formation from the parent ion.

In the case of *cis*-bis(methylthio)silvatin (**1**), based on the known composition of its parent ion and its isotopic pattern, the formula C_14_H_17_N_2_O_3_S^+^ can be attributed to this ion, which implies a loss of C_6_H_11_S from the molecular ion. This loss is interpreted structurally as shown in Scheme 3.

The loss of C_6_H_11_S from the molecular ion of metabolite **2** would create the ion C_13_H_15_N_2_O_3_S^+^ at *m*/*z* = (394−115) = 279, which is not the case.

In Scheme 3, it is shown that the shared ion C_14_H_17_N_2_O_3_S^+^ (*m*/*z* = 293) can be created from the hypothesized structure of metabolite **2** by a loss of C_5_H_9_S, instead, which is produced by moving scission of the σ bond from the allylic position (in the case of *cis*-bis(methylthio)silvatin (**1**)) to the C-C σ bond, starting at the double bond of the alkyl side chain.

The two-bond scission which, from *cis*-bis(methylthio)silvatin (**1**), produces the ion at *m*/*z* 293, takes place in two steps. In the first step, only one of the two CH_3_S- groups is eliminated and this produces the well-developed peak at *m*/*z* 361 (i.e., C_19_H_25_N_2_O_3_S^+^) in spectrum (A). The formed ion then evolves to the shared ion C_14_H_17_N_2_O_3_S^+^ (*m*/*z* = 293) by dissociation of the alkyl chain bonded to the phenolic oxygen atom.

On the contrary, there is no signal at *m*/*z* = 394 - 47 = 347 due to dissociation of the CH_3_S- group (which would produce the ion C_18_H_23_N_2_O_3_S^+^) in the mass spectrum of metabolite **2**, but the intensity of the peak at *m*/*z* 293 is about three times the intensity of the corresponding peak in the spectrum of *cis*-bis(methylthio)silvatin (**1**). This simply implies that the intermediate ion C_18_H_23_N_2_O_3_S^+^ (*m*/*z* = 347) produced from metabolite **2** evolves, after its formation in the ion source, with a larger speed than ion C_19_H_25_N_2_O_3_S^+^ (*m*/*z* = 361), to the shared ion C_14_H_17_N_2_O_3_S^+^ (*m*/*z* = 292); so much so that the abundance lost at *m*/*z* = 347 in spectrum (B) is fully recovered as an increase of abundance at *m*/*z* 293. This explains the fundamental qualitative difference between the spectra in Figure 5.

For brevity, we omit further correlations which can be stated between the mass spectra in Figure 5 and which support the hypothesized structure of metabolite **2** exposed in Figure 4.

We also mention that when the spectrum of *cis*-bis(methylthio)silvatin (**1**) showed in Figure 5 is submitted, along with the known structure, to the algorithm of MS Interpreter, as expected, about 85% of the total ionic abundance (i.e., 7078) in the spectrum is explained. The analogous operation performed with the mass spectrum of metabolite **2** and its hypothetical structure results in 83.8% of the total ionic abundance (i.e., 6322) being explained.

As anticipated above, the response of MS Interpreter was the same, regardless of which of structures **2**A and **2**B in Figure 4 is submitted, implying that it is impossible to distinguish between these two isomers.

Finally, as a matter of fact, structures **2**A and **2**B in Figure 4 correspond to isomers A/B of saroclazine which are known fungal metabolites [14]. Then, it appears very reasonable to identify metabolite **2** with saroclazine A/B.

In this way, as sketched in the supplementary materials, we have arrived at structures of compounds in Table 1 which could not be recovered in a sufficient quantity and/or at a suitable degree of purity for NMR analysis.

Several thousand secondary metabolites have been isolated from fungi. Depending on their chemical structure, fungal metabolites can display beneficial or harmful effects on human health. In fact, many have been reported to possess a broad range of biological activities (including antitumor, antiviral, antimicrobial, anti-inflammatory, and antioxidant effects), and have thus been used as lead compounds in medicine. However, a lot of fungal metabolites with negative effects on human health (for example, mycotoxins) have also been identified. In this study, two main secondary metabolites were purified from the culture filtrate of strain AN4, which are typical products of the species *P. brevicompactum*. In particular, mycophenolic acid is considered to characterize the metabolomic profile of this species so that, together with brevianamide A, which was also detected through LC-MS and GC (metabolite **9** in Table 1), it is regarded as a chemotaxonomic marker [15,16]. Conversely, asperphenamate, which is another product typical of all the species in the section Brevicompacta [17], was not detected in our study. The second purified product, cis-bis(methylthio)silvatin, is a member of thiosilvatins, a homogeneous family of compounds within the epipolythiodioxopiperazines, which, so far, have been reported from just 22 strains belonging to 17 fungal species from both marine and terrestrial sources. Half of these strains belong to *Penicillium* species, indicating that the genetic basis for thiosilvatin biosynthesis may be rooted in this genus [12]. Despite *P. brevicompactum* being very frequently recovered from many ecological contexts, this product has already been reported from just a single non-marine strain of this species, together with deprenyl-bis(methylthio)silvatin [18], indicative of either its infrequent occurrence or inherent difficulties in extraction and/or identification. Conversely, the other thiosilvatins identified in this study (i.e., **2-5** and **8**) represent new records for *P. brevicompactum*. *Cis*-bis(methylthio)silvatin was previously reported for antibacterial activity against *Staphylococcus aureus* and, more importantly, its cytotoxic effect against P388 lymphocytic leukemia and NS-1 cells [19]. For the first time, we have investigated the effect of cis-bis(methylthio)silvatin on two colorectal adenocarcinoma cell lines with a different genetic profile; in fact, HCT116 expresses mutant β-catenin and K-RAS, while Caco-2 exhibits APC*,* β-catenin, and p53 gene mutations [20]. In comparison, a non-tumoral colonic epithelial cell line was assayed. Our results have shown that the compound exerts a cytotoxic effect on the colon cancer cell lines with a similar efficacy and potency, but without substantial selectivity toward the non-tumoral cell line.

## 4. Materials and Methods

### 4.1. General Experimental Procedures

Optical rotations were measured in CHCl_3_ using a Jasco P-1010 digital polarimeter (Tokyo, Japan). ^1^H and ^13^C NMR spectra were recorded at 400/100 MHz in CDCl_3_ on a Bruker spectrometer (Ascend^TM^400) (Bremen, Germany). The same solvent was used as the internal standard. (COSY)-45 experiments were performed using standard Bruker microprograms [21]. Analytical and preparative TLC were performed on silica gel plates (Kieselgel 60, F_254_, 0.25 mm) purchased from Merck (Darmstadt, Germany). The spots were visualized by exposure to UV radiation (253 nm), or by spraying with 10% H_2_SO_4_ in MeOH, followed by heating at 110 °C for 10 min. Column chromatography (CC) was performed using silica gel (Kieselgel 60, 0.063–0.200 mm) purchased from Merck.

### 4.2. Isolation and Identification of Strain AN4

Strain AN4 was recovered from a Mediterranean snakelocks sea anemone (*A. sulcata*) collected in the intertidal zone along the coastline of the isle of Procida (Naples Bay, Italy) by plating a tissue fragment excised from the pedal disc on potato dextrose agar (PDA) amended with streptomycin sulphate (200 mg L^−1^). The fungal isolate was transferred in pure culture and stored at 4 °C. Subcultures were prepared on Czapek-Dox agar (CDA) and malt extract agar (MEA) for morphological observations. The strain formed slow growing velutinous colonies, which turned dull green as sporulation progressed. Finely roughened ellipsoidal conidia were produced on short terverticillate conidiophores, with all elements adpressed, which were clearly indicative of its belonging in the section Brevicompacta of the genus *Penicillium* [17,22]. For a more circumstantial species ascription, the translation elongation factor 1-alpha (TEF) gene sequence was extracted according to a previously published procedure [23], which showed a 100% homology with TEF sequences of a few strains of *P. brevicompactum* available in GenBank, including the type strain NRRL2011. Our sequence has in turn been deposited in GenBank under the code MN548283.

### 4.3. Production and Fractionation of Culture Filtrates

Mycelial plugs from actively growing cultures of strain AN4 were inoculated in 1 L Erlenmayer flasks containing 500 mL of Czapek-Dox broth (Oxoid, Hampshire, UK). The liquid cultures (1 L) were incubated in the dark at 25 ± 2 °C on stationary phase. After 14 days, the fungal biomass was removed through filtration (Whatman No. 4 filter paper), and the culture filtrate was extracted at a native pH (4.2) with ethyl acetate. The organic extracts were combined, dried with Na_2_SO_4_, and evaporated under reduced pressure at 37 °C to give a yellow oily residue (29.4 mg). The crude organic extract was submitted to fractionation on a silica gel column (1.0 × 40 cm ID), eluted with CHCl_3_/iso-PrOH (95:5). The last fraction was eluted with MeOH. Six homogeneous fractions were collected and pooled on the basis of similar TLC profiles (A 1.6, B 5.2, C 1.5, D 1.6, E 7.2, and F 11.9 mg). The residue of fraction B after purification by TLC on silica gel eluted with n-hexane/ethyl acetate (6:4, *v/v*), produced the yellow solid identified as **1** (Figure 1, 3.5 mg, R_f_ 0.85). The residue from fraction E was further purified by preparative TLC on silica gel eluted with CHCl_3_/i-PrOH (92:8, *v/v*), producing mycophenolic acid (**8**, Figure 1, 5.8 mg, R_f_ 0.48).

### 4.4. LC-MS Analysis

Analyses were conducted on an Agilent HP 1260 Infinity Series liquid chromatography equipped with a DAD system coupled to a Q-TOF mass spectrometer (Agilent Technologies, Santa Clara, CA, USA). An Ascentis^®^ Express C18 column (3.0 × 50 mm, 2.7 µm, Supelco, Bellefonte, PA, USA) was used for chromatographic separation. The analyses were performed at a 0.4 mL min^−1^ flow rate and a constant temperature of 37 °C, using a linear gradient system composed of 0.1% (*v/v*) formic acid in water (eluent A), and 0.1% (v/v) formic acid in acetonitrile (eluent B). The gradient program was as follows: from 5% to 100% eluent B in 6 min, isocratic at 100% of eluent B from 6 to 8 min, and from 100% to 5% eluent B from 8 to 10 min. After returning to the initial conditions, equilibration was achieved after 2 min. The injection volume was 7 µL [24,25,26].

The UV spectra were collected by DAD every 0.4 s from 190 to 750 nm, with a resolution of 2 nm. The MS system was equipped with a Dual Electrospray Ionization (ESI) source and operated with Agilent MassHunter Data Acquisition Software, rev. B.05.01, in the positive mode. Mass spectra were recorded within the *m*/*z* range 50–1700 as centroid spectra, with three scans per second. The mass spectrometer was calibrated using the ESI-L Low Concentration Tuning Mix (Agilent Technologies). Additionally, real-time lock mass correction was performed using purine (C_5_H_4_N_4_ at *m*/*z* 121.050873, 10 µmol L^−1^) and hexakis (^1^H,^1^H,3H-tetrafluoropentoxy)-phosphazene (C_18_H_18_O_6_N_3_P_3_F_24_ at *m*/*z* 922.009798, 2 µmol L^−1^), according to the manufacturer’s instructions (Agilent Technologies). Solvents were LC–MS grade, and all other chemicals were analytical grade, purchased from Sigma-Aldrich (Saint Louis, MO, USA) (unless otherwise stated).

Data were evaluated using MassHunter Qualitative Analysis Software B.06.00 and comparisons with known compounds were made in an in-house database combined with data from the literature. Positive identifications of fungal metabolites were considered for analysis if the compound was detected with a mass error below 10 ppm and with a sufficient score.

### 4.5. GC-MS Analysis

GC-MS measurements were performed with an Agilent 6850 GC equipped with an HP-5MS capillary column (5% phenyl methyl polysiloxane stationary phase) and the Agilent 5973 Inert MS detector (used in the scan mode). Helium was employed as the carrier gas, at a flow rate of 1 mL min^−1^. The injector temperature was 250 °C and during the run, a temperature ramp raised the column temperature from 70 to 280 °C: 70 °C for 1 min; 10 °C min^−1^ until reaching 170 and 30 °C min^−1^ until reaching 280 °C; 280 °C for 20 min. The electron impact (EI) ion source was operated at 70 eV and 200 °C. The quadrupole mass filter was kept at 250 °C and was programmed to scan the range 45–550 *m*/*z* at a frequency of 3.9 Hz. A crude extract was analyzed both directly and after trimethylsilylation with BSTFA, as described in [27]. Metabolites were identified by interpreting their mass spectra via MS interpreter v3.4 distributed by the National Institute of Standards and Technology (NIST) [28].

*Cis-bis(methylthio)silvatin* (**1**). [α]_D_^25^ -47.5 (CHCl_3_, *c* 0.2); HR ESI MS (+) spectrum *m*/*z*: 839.2969 [C_40_H_56_N_4_O_6_S_4_Na, calcd. 839.2980, 2M+Na]^+^, 431.1433 [C_20_H_28_N_2_O_3_S_2_Na, calcd. 431.1439, M + Na]^+^, 361.1579 [C_19_H_25_N_2_O_3_S, calcd. 361.1586, M-SCH_3_]^+^. GC-MS data: RI 3229; *m*/*z*: 361 [M-CH_3_S]^+^ (26), 293 [M-C_6_H_11_S]^+^ (23), 245 [M-C_5_H_8_NO_2_S-H_2_O]^+^ (46), 233 [M-C_12_H_16_O]^+^ (60), 217 [M-C_13_H_21_O]^+^ (21), 186 [M-C_13_H_19_OS]^+^ (40), 107 [M-C_13_H_21_N_2_O_2_S_2_]^+^ (100).

*Saroclazine A/B* (**2**). HR ESI MS (+) spectrum *m*/*z*: 395.1623 [C_19_H_27_N_2_O_3_S_2_, calcd. 251.1278, M + H]^+^. GC-MS data: RI 2892, 340 [M-C_4_H_7_]^+^ (3), 293 [M-C_6_H_14_O]^+^(76), 245 [M-C_4_H_8_N_2_OS-H_2_O]^+^ (28), 235 [M-C_11_H_14_N_2_O]^+^ (35), 218 [M-C_11_H_14_O-CH_3_]^+^ (33), 187 [M-C_12_H_20_N_2_O_2_S_2_]^+^ (100).

*Fusaperazine E/F* (**3**). HR ESI MS (+) spectrum *m*/*z*: 383.1822 [C_19_H_24_O_3_N_2_SNa, calcd. 383.1405, M + Na]^+^, 361.1574 [C_19_H_25_O_3_N_2_SNa, calcd. 361.1586, M+H]^+^. GC-MS data: RI 2083, 361 [M]^+^ (100), 312 [M-CH_4_S]^+^ (47), 234 [M-C_7_H_13_NO]^+^ (52), 187 [M-C_8_H_16_NOS]^+^ (76).

*6-Oxo-methylthiosilvatin* (**4**). HR ESI MS (+) spectrum *m*/*z*: 775.2817 [C_38_H_48_N_4_O_8_S_2_Na, calcd. 775.2811, 2M + Na]^+^, 399.1354 [C_19_H_24_N_2_O_4_SNa, calcd. 399.1345, M+Na]^+^, 377.1528 [C_19_H_25_N_2_O_4_S, calcd. 377.1535, M+H]^+^, 361.1575 [M-CH_3_calcd. 361.1222]^+^. GC-MS data: RI 3203, *m*/*z*: 360 [M-CH_4_]^+^ (4), 292 [M-C_5_H_8_O]^+^ (23), 245 [M-C_4_H_4_NO_3_-H_2_O]^+^ (100), 217 [M-C_5_H_5_NO_3_S]^+^ (44), 146 [M-C_8_H_12_N_2_O_3_S-CH_3_]^+^ (36).

*Bilain B* (**5**). HR ESI MS (+) spectrum *m*/*z*: 443.3332 [C_20_H_31_N_2_O_5_S_2_, calcd. 443.1674, M+H]^+^. GC-MS data (silylil derivative): RI 3047, 280 [M-C_13_H_31_SSi_2_]^+^ (50), 218 [M-C_15_H_18_N_2_O_2_S_2_-H_2_O]^+^ (25).

*Deprenyl-bis(methylthio)silvatin* (**6**). HR ESI MS (+) spectrum *m*/*z*: 363.3094 [C_15_H_20_O_3_N_2_O_3_S_2_Na, calcd. 363.0815, M+Na]^+^, 341.2656 [C_15_H_21_O_3_N_2_O_3_S_2_, 341.4688, M+H]^+^. GC-MS data (silylil derivative): RI 2014, 412 [M]^+^ (25), 369 [M-C_2_H_4_O]^+^ (25), 321 [M-C_3_H_10_S-H_2_O]^+^ (47), 294 [M-C_4_H_9_NOS]^+^ (12).

*Fusaperazine A* (**7**). HR ESI MS (+) spectrum *m*/*z*: 335.2791 [C_13_H_16_N_2_O_3_S_2_Na, calcd. 335.2804, M+Na]^+^, 313.2351 [C_13_H_17_N_2_O_3_S_2_, calcd. 313.2278, M+H]^+^. GC-MS data (silylil derivative): RI 1876, 384 [M]^+^ (5), 369 [M-CH_3_]^+^ (100), 353 [M-2(CH_3_)]^+^ (5), 295 [M-C_3_H_8_Si-H_2_O]^+^ (12), 237 [M-C_4_H_7_NO_2_S-CH_3_]^+^ (10).

*Mycophenolic acid* (**8**). HR ESI MS (+) spectrum *m*/*z*: 663.24092 [C34H40O12Na, calcd. 663.2417, 2M+Na]^+^, 343.1153 [C_17_H_20_O_6_Na, calcd. 343.1158, M+Na]^+^, 321.1331 [C_17_H_21_O_6_, calcd. 321.1338, M+H]^+^, 303.1228 [C_17_H_19_O_5_, calcd. 303.1232, M-OH]^+^. GC-MS data: RI 2871, *m*/*z*: 320 [M]^+^ (19), 302 [M-H_2_O]^+^ (15), 247 [M-C_3_H_6_O_2_]^+^ (57), 207 [M-C_6_H_10_O_2_]^+^ (100).

*Brevianamide A/B* (**9**). HR ESI MS (+) spectrum *m*/*z*: 753.3359 [C_42_H_46_O_6_N_6_Na, calcd. 753.3356, 2M + Na]^+^, 731.3549 [C_42_H_47_O_6_N_6_, calcd. 753.3557, 2M + H]^+^, 388.1620 [C_21_H_23_O_3_N_3_Na, calcd. 388.1637, M + Na]^+^, 366.1810 [C_21_H_24_O_3_N_3_, calcd. 366.1818, M+H]^+^. GC-MS data (silylil derivative): RI 3227, 361 [M-C_6_H_5_]^+^ (45), 245 [M-C_9_H_10_N_2_O_2-_CH_3_-CH_3_]^+^ (48), 233 [M-C_11_H_15_NOS]^+^ (73), 217 [M-C_12_H_17_N_2_O_2_]^+^ (18), 186 [M-C_12_H_20_N_2_O_2_Si]^+^ (50).

### 4.6. Bioassay

#### 4.6.1. Cell Lines

For in vitro experiments, two human colon adenocarcinoma cell lines (i.e., Caco-2 and HCT116, ATCC from LGC Standards, Milan, Italy) and a non-tumoral colonic epithelial cell (HCEC, a kind gift of Fondazione Callerio Onlus, Trieste, Italy) were used. Caco-2 and HCT116 were cultured in Dulbecco’s modified Eagle’s medium (DMEM) containing 10% fetal bovine serum (FBS), 100 U mL^−1^ penicillin, and 100 μg mL^−1^ streptomycin; 1% non-essential amino acids; 2 mM l-glutamine; and 1 M HEPES, in conformity with the manufacturer’s protocols. The immortalized HCEC, derived from human colon biopsies and used as a comparison with tumor cells, was cultured in DMEM supplemented with 10% FBS, 100 U mL^−1^ penicillin, 100 μg mL^−1^ streptomycin, 200 mM l-glutamine, 100 mM Na-pyruvate, and 1 M HEPES.

#### 4.6.2. Cell Viability Assay

Cell viability was evaluated by measuring the mitochondrial reductase activity (MTT assay). Cells were seeded in the presence of 10% FBS in 96-well plates at a density of 10^4^ cells per well and allowed to adhere for 24 h. After this period, cells were incubated with medium containing 10% FBS in the presence or absence of increasing concentrations of *cis*-bis(methylthio)silvatin (0.3 ÷ 100 μM, previously dissolved in DMSO 0.1%) or DMSO 20% used as a positive control, for 24 h. Subsequently, the treatment medium was replaced with fresh medium containing MTT (250 μg mL^−1^, for 1 h at 37 °C). After solubilization in DMSO, the mitochondrial reduction of MTT to formazan was quantitated at 490 nm (iMarkTM microplate reader, Bio-Rad). All results are expressed as the percentage of cell viability.

#### 4.6.3. Statistical Analysis

Data are expressed as the mean ± SEM of three experiments. Statistical analysis was performed with the software GraphPad Prism (ver. 7.00) using one-way ANOVA, followed by a Tukey multiple comparisons test (for the analysis of multiple treatment means). EC_50_ values were calculated by non-linear regression analysis using the equation for a sigmoid concentration–response curve (GraphPad Prism). P-value < 0.05 was considered to be significant.

## 5. Conclusions

Our metabolomic analysis, carried out by combining several instrumental techniques and structural elucidation approaches, disclosed the ability by strain AN4 of *P. brevicompactum* to produce several compounds belonging to the thiosilvatin series [12], in evident connection with their biosynthetic relationships. Seven compounds in this series, of which five were not previously reported in *P. brevicompactum*, were detected as secondary metabolites of strain AN4.

## Figures and Tables

**Table 1:** Data concerning nine targeted metabolites of strain AN4 collected via GC-EI-MS and LC-MS ESI-HRMS. Uncertainty with regards to the reported values of the accurate mass is typically ±0.0002–0.0005 u. RDB (Ring + Double Bonds) is calculated from empirical formulas by a standard algorithm [11].

Code	Compound	GC-EI-MS Data and RI	Formula	Accurate Mass (u)	RDB
**1**	*cis*-bis(methylthio)silvatin	Compound (RI = 3229)	C_20_H_28_N_2_O_3_S_2_	408.1538	8
**2**	Metabolite 2	Compound (RI = 2892)	C_19_H_26_N_2_O_3_S_2_	394.1388	8
**3**	Metabolite 3	Compound (RI = 2083)	C_19_H_24_N_2_O_3_S	360.1510	9
**4**	Metabolite 4	Compound (RI = 3203)	C_19_H_24_N_2_O_4_S	376.1462	9
**5**	Metabolite 5	TMS derivative (RI = 3047)	C_20_H_30_N_2_O_5_S_2_	442.1593	7
**6**	Metabolite 6	TMS derivative (RI = 2014)	C_15_H_20_N_2_O_3_S_2_	340.0919	7
**7**	Metabolite 7	TMS derivative (RI = 1876)	C_13_H_16_N_2_O_3_S_2_	312.0600	7
**8**	mycophenolic acid	Compound (RI = 2871)	C_17_H_20_O_6_	320.1268	8
**9**	Metabolite 9	TMS derivative (RI = 3227)	C_21_H_23_N_3_O_3_	365.1725	12

## Supplementary Materials

The following are available online at https://www.mdpi.com/2218-1989/10/2/55/s1. Metabolite identification, Figure S1. LC-MS chromatogram of crude extract, Figures S2–S6. NMR spectra of *cis*-bis(methylthio)silvatin and mycophenolic acid, Figure S7. EI mass spectra at 70 eV of **1**–**9**, Figures S8–S15. ESI-MS mass spectra of **1**–**9**.
