# Supplementary material for: Identification of the Main Metabolites of a Marine-Derived Strain of Penicillium brevicompactum Using LC and GC MS Techniques
Source: Metabolites. 2020 Jan 30;10(2):55. doi: 10.3390/metabo10020055 (PMC7074196; doi:10.3390/metabo10020055)
Supplement: Supplementary file 1 [file metabolites-10-00055-s001.pdf]

Supplementary Materials

Identification of Main Metabolites of a Marine-derived Strain of *Penicillium brevicompactum* using LC and GC MS Techniques

Francesco Vinale[1,2], Maria Michela Salvatore[3], Rosario Nicoletti[4,5], Alessia Staropoli[2,5], Gelsomina Manganiello[5], Tommaso Venneri[6], Francesca Borrelli[6], Marina DellaGreca[3], Francesco Salvatore [3] and Anna Andolfi[3,*]

1 Department of Veterinary Medicine and Animal Productions, University of Naples Federico II, 80137 Naples, Italy; frvinale@unina.it(F.V.)

2 Institute for Sustainable Plant Protection, National Research Council, 80055 Portici (NA), Italy; (F.V.), alessia.staropoli@unina.it (A.S.);

3 Department of Chemical Sciences, University of Naples Federico II, 80126 Naples, Italy; mariamichela.salvatore@unina.it (M.M.S.); dellagre@unina.it(M.D.); frsalvat@unina.it(F.S.)

4 Council for Agricultural Research and Economics, Research Centre for Olive, Citrus and Tree Fruit, 81100 Caserta, Italy; rosario.nicoletti@crea.gov.it(R.N.)

5 Department of Agricultural Sciences, University of Naples Federico II, 80055 Portici (NA), Italy; gelsomina.manganiello@unina.it(G.M.)

6 Department of Pharmacy, School of Medicine and Surgery, University of Naples Federico II, 80131 Naples, Italy; tommaso.venneri@unina.it(T.V); franborr@unina.it(F.B.)

* Correspondence: andolfi@unina.it; Tel.: +39-081-2539179

Received: 17 December 2019; Accepted: 27 January 2020; Published: date

Metabolite identification

Metabolite structures reported in figure 1 were assigned following the same way used for metabolite **2**. Here the identification of each metabolite is explained in detail.

In particular, formula of metabolite **3** ($C_{19}H_{26}N_2O_3S_2$) can be obtained from **1** by elimination of one molecule of methanethiole (CH_3SH) with the simultaneous formation of a double bond. Based on this observation structure of **1** is easily converted in the structure of metabolite **3** in Figure 1, which is strongly supported by the interpretation of the EI mass spectrum via MS Interpreter. Apart from that, a search of PubChem database for the structure assigned to metabolite **3**, results in a structure identified by the PubChem CID 101520130 (common name: Fusaperazine E).

Conversely, the case of metabolite **4** could not unambiguously be resolved in this way. In fact, from one side, metabolite **4** appears to be formed in the same way as metabolite **3** by elimination of a methanethiole molecule from the formula of **1**. As in the case of metabolite **3**, this justifies the fact that this molecule has one C, four H and one S atom less than **1**, and a one unit higher RDB number. However, this does not justify the fact that metabolite **4** exposes one more oxygen atom than **1**. To solve this problem, we performed a structure similarity search in PubChem by starting with the SMILES string of **1**. This search resulted in a list of 61 compounds which, according to the PubChem search algorithm, had structures related to **1**. By parsing this list, we found a single structure whose formula ($C_{19}H_{24}N_2O_4S$) coincides with the one assigned to metabolite **4**. This structure is identified by the PubChem CID 122229044 and the IUPAC name: (6*S*)-1,4-dimethyl-6-[[4-(3-methylbut-2-enoxy)phenyl]methyl]-6-methylsulfanylpiperazine-2,3,5-trione. The structure of this compound was readily downloded from the PubChem site and imported in MS Interpreter with the experimental mass spectrum associated to metabolite **4**. This showed a very high degree of consistency between the downloaded structure and the mass spectrum; thus, structure presented in Figure 1 was assigned to metabolite **4**.

Now we consider metabolites **5**, **6** and **7** in the third column of Figure 3. All these metabolites share the same RDB which is one unit less than the RDB number of **1**. It is most reasonable to start with the idea that this RDB decrease takes place at the expenses of the double bond in the alkyl side chain attached to the benzene ring in the structure of **1**. This idea is readily confirmed by parsing the above mentioned PubChem list of structures similar to **1** for the formula $C_{20}H_{30}N_2O_5S_2$, associated to metabolite **5**. A single structure is associated to this formula, identified by the PubChem CID 24179621 (common name: bilain B). The structure of bilain B (Figure 1) is formally derived from that of **1** by converting the double C=C bond of the alkyl side chain in a diol functional group. This beautifully justifies the decrease in RDB, and the increase in O and H atoms in passing from the formula of **1** to $C_{20}H_{30}N_2O_5S_2$.As in the previous case, we obtained a positive response when the structure of the bis TMS derivative of bilain B was compared, via MS Interpreter, to the EI Mass spectrum associated to metabolite **5**.

In the above PubChem list there was no structure associated to the formula of metabolite **6**, but there was a structure associated to the formula of metabolite **7**. This structure, identified by the PubChem CID 10267534 (common name: fusaperazine A), is exposed in Figure 1. The structure of fusaperazine A can be derived from structure of **1** by the simultaneous substitution of the $-C_5H_9$ side chain and of the two $-CH_3$ methyl groups, bonded to the N atoms of the heterocyclic ring, with H atoms. This beautifully accounts for the decrease in the number of C and H atoms and RDB in passing from the formula of **1** ($C_{20}H_{28}N_2O_3S_2$) to the formula associated to metabolite **7** ($C_{13}H_{16}N_2O_3S_2$). As in previous cases, the structure of the TMS derivative of fusaperazine A was found to explain satisfactorily the mass spectrum associated with metabolite **7** using MS Interpreter and our own knowledge of fragmentation rules.

In the light of the way in which the structure of fusaperazine A is connected to the structure of **1**, the structure of metabolite **6**, can easily be inferred. In fact, the formula of metabolite **6** ($C_{15}H_{20}N_2O_3S$) can easily be derived from **1** through a path in which (as in fusaperazine A) the alkyl side chain, $-C_5H_9$, is disconnected from the benzene ring and substituted with one H atom. The difference between this path and the path leading from **1** to fusaperazine A simply consists in the fact that the $-CH_3$ groups attached to the nitrogen atoms are left unchanged. From this inference a structure is created which corresponds to the Simplified Molecular Input Line Entry Specification string (SMILES) <CN1C(C(=O)N(C(C1=O)(CC2=CC=C(C=C2)O)SC)C)SC>. When this SMILES string is searched in the PubChem database, the searching algorithm readily finds a structure identified by the PubChem CID 13989134 and the IUPAC name (3*R*,6*R*)-3-[(4-hydroxyphenyl)methyl]-1,4-

dimethyl-3,6-bis(methylsulfanyl)piperazine-2,5-dione. After checking for its consistency with the mass spectrum associated to metabolite **6**, the above structure was definitely attributed to metabolite **6** (Figure 1).

Please note that structures of metabolites **4**, **5**, **6** and **7** reported in Figure 1 refers to the stereoisomer indexed in PubChem database but, since mass spectrometry cannot distinguish between stereoisomers, it is strictly non-significant.

As it can be easily predicted from its formula ($C_{21}H_{23}N_3O_3$) and from the higher value of RDB (RDB = 13), the above approach cannot be extended to metabolite **9** in Table 1. Furthermore, a blind search in the PubChem database is immaterial because it finds thousands of results corresponding to formula $C_{21}H_{23}N_3O_3$. Nonetheless, a mycotoxin whose formula is $C_{21}H_{23}N_3O_3$, named brevianamide A (CAS#: 23402-09-7), is a well-known product of *Penicillium brevicompactum* and some other *Penicillium* species. Brevianamide A is an aromatic heteropolycyclic compound which, as predictable from its structure, can be silylated at least at one of the three nitrogen atoms. This is in agreement with the fact that the mass spectrum associated with metabolite **9** corresponds to its TMS derivative. When the structure of the TMS derivative of brevianamide A is imported in MS Interpreter, a conspicuous number of peaks in the experimental mass spectrum are compatible, although several mass peaks are not predicted and are coloured in white. This is to be expected in view of the structural complexity of brevianamide A which makes the prediction of fragmentation paths a difficult task. Thus, metabolite **9** is identified with brevianamide A (or its conformer brevianamide B, CAS#: 38136-92-4), whose structure can be seen in Figure 1.

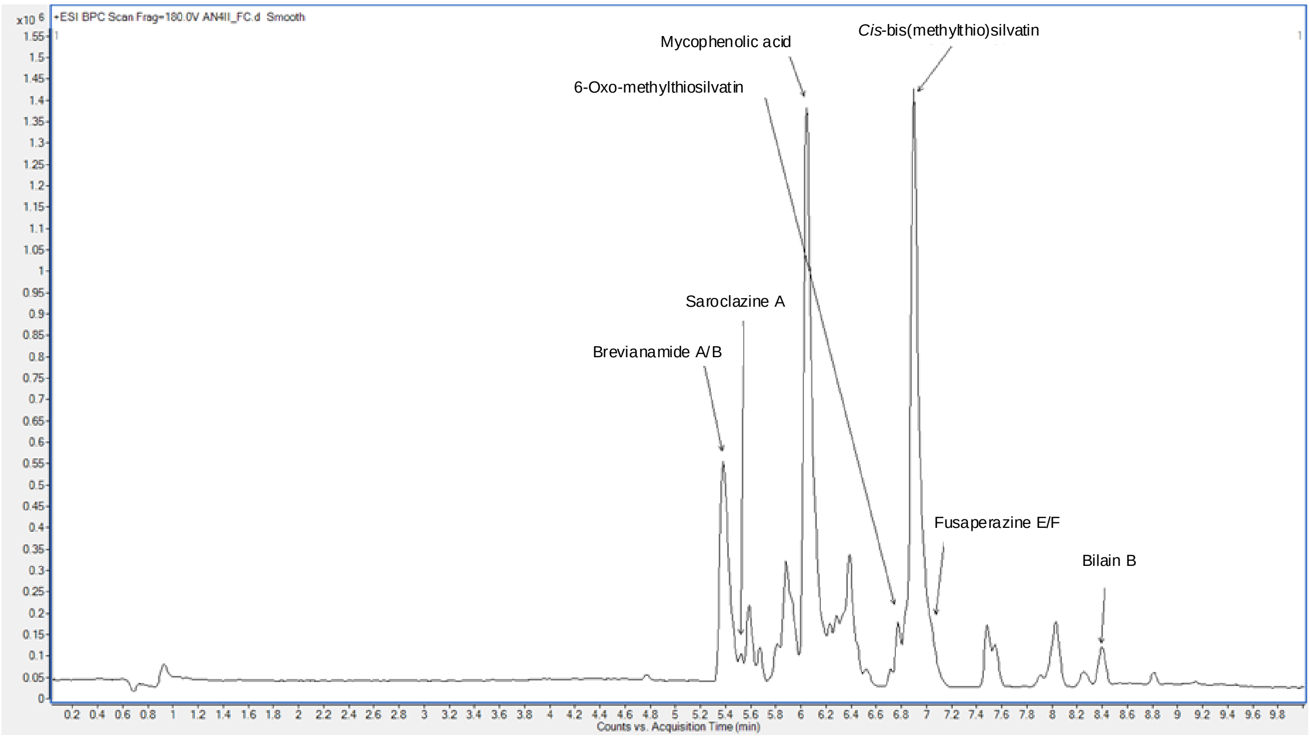

Figure S1. LC-ESI-HRMS chromatogram of crude extract of *Penicillium brevicompactum* (AN4) recorded in positive mode.

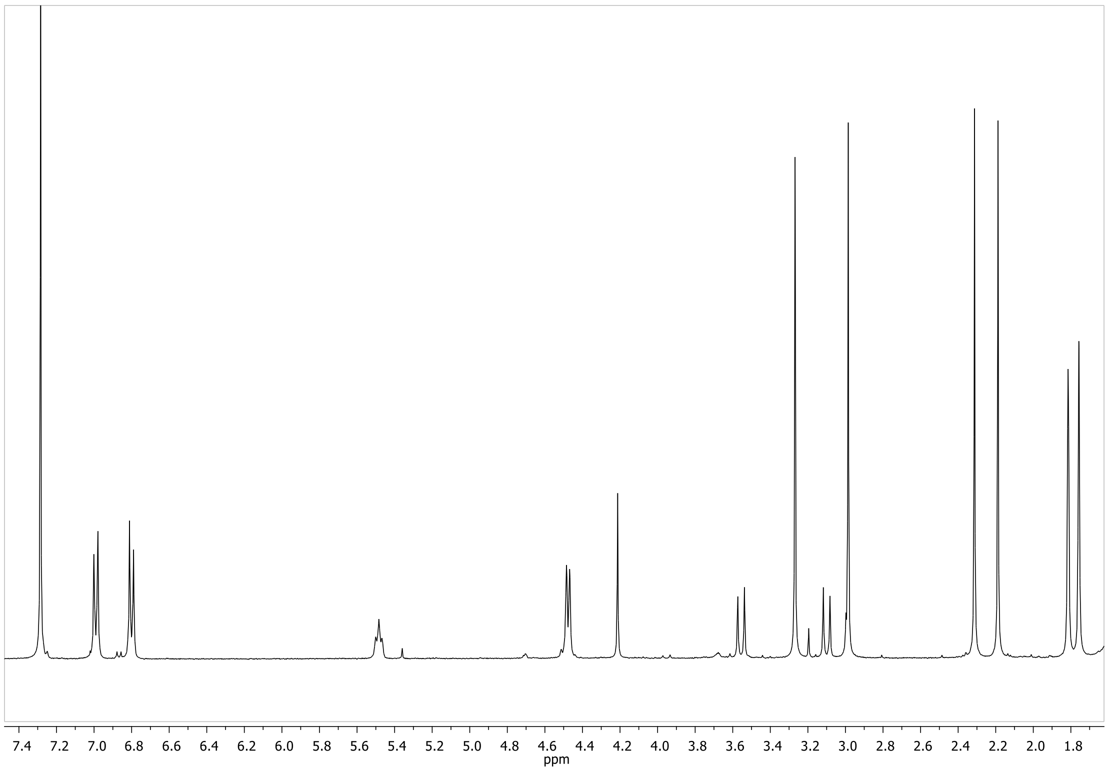

Figure S2. ^{1}H NMR spectrum of *cis*-bis(methylthio)silvatin (**1**) recorded in $CDCl_3$ at 400 MHz.

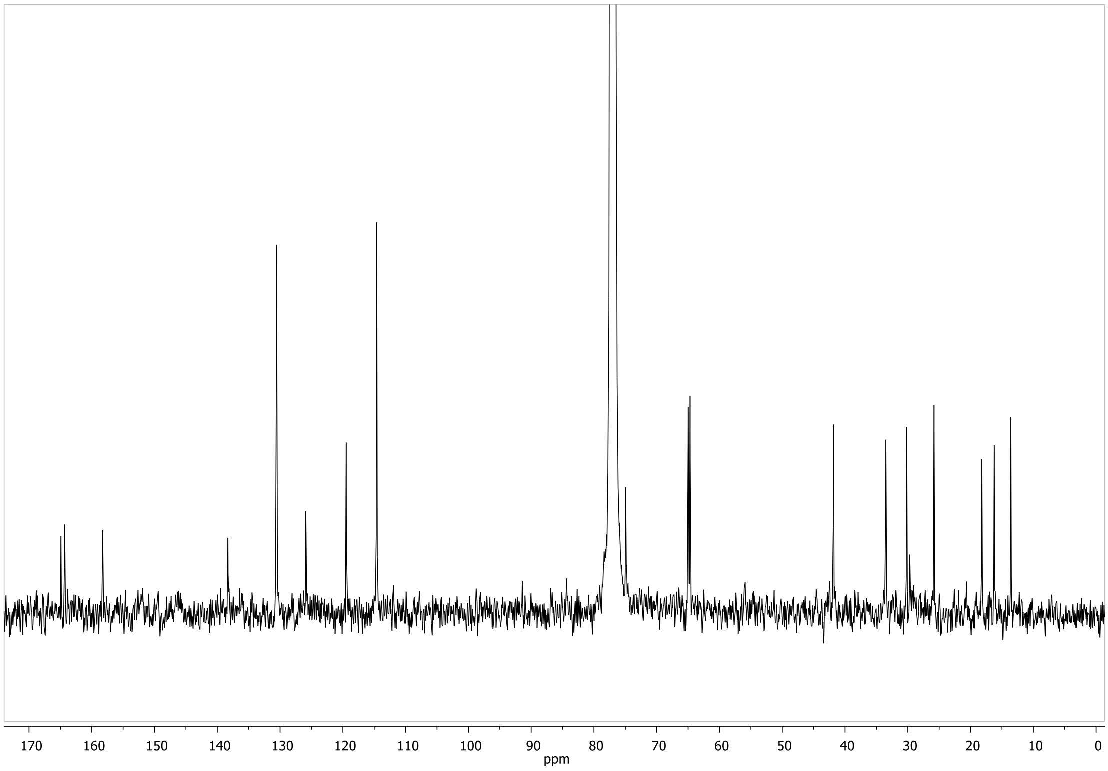

Figure S3. ^{13}C NMR spectrum of *cis*-bis(methylthio)silvatin (**1**) recorded in $CDCl_3$ at 100 MHz.

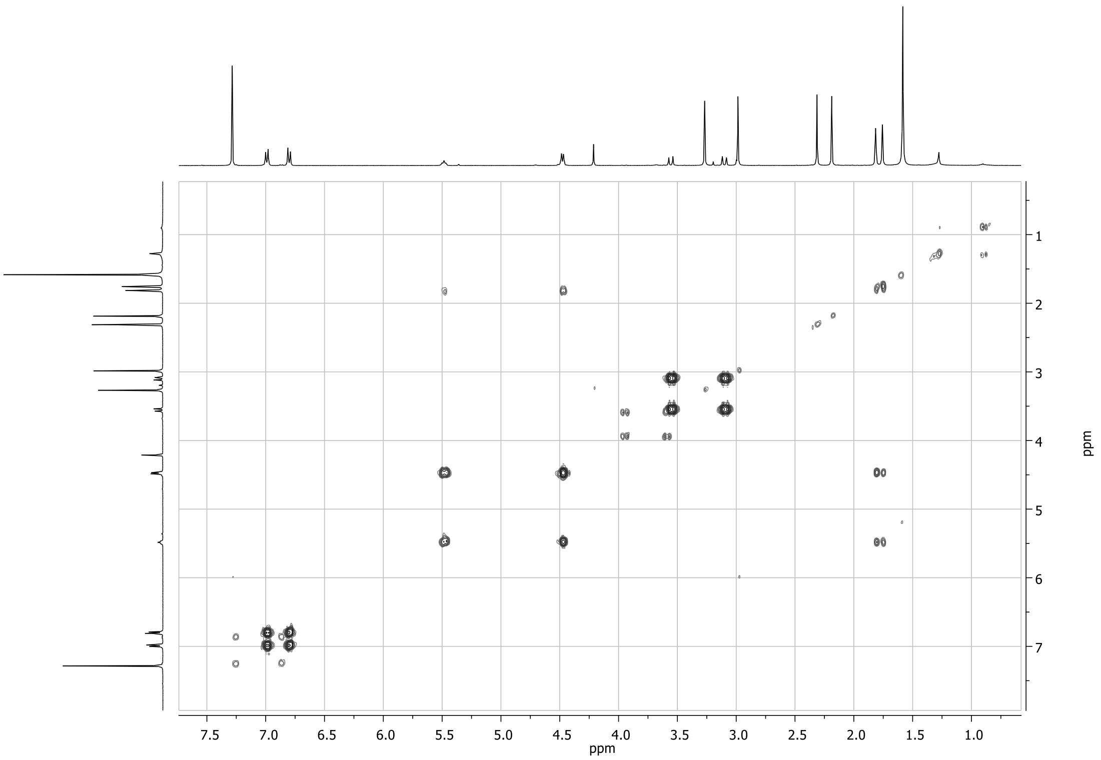

Figure S4. COSY spectrum of *cis*-bis(methylthio)silvatin (**1**) recorded in $CDCl_3$ at 400 MHz.

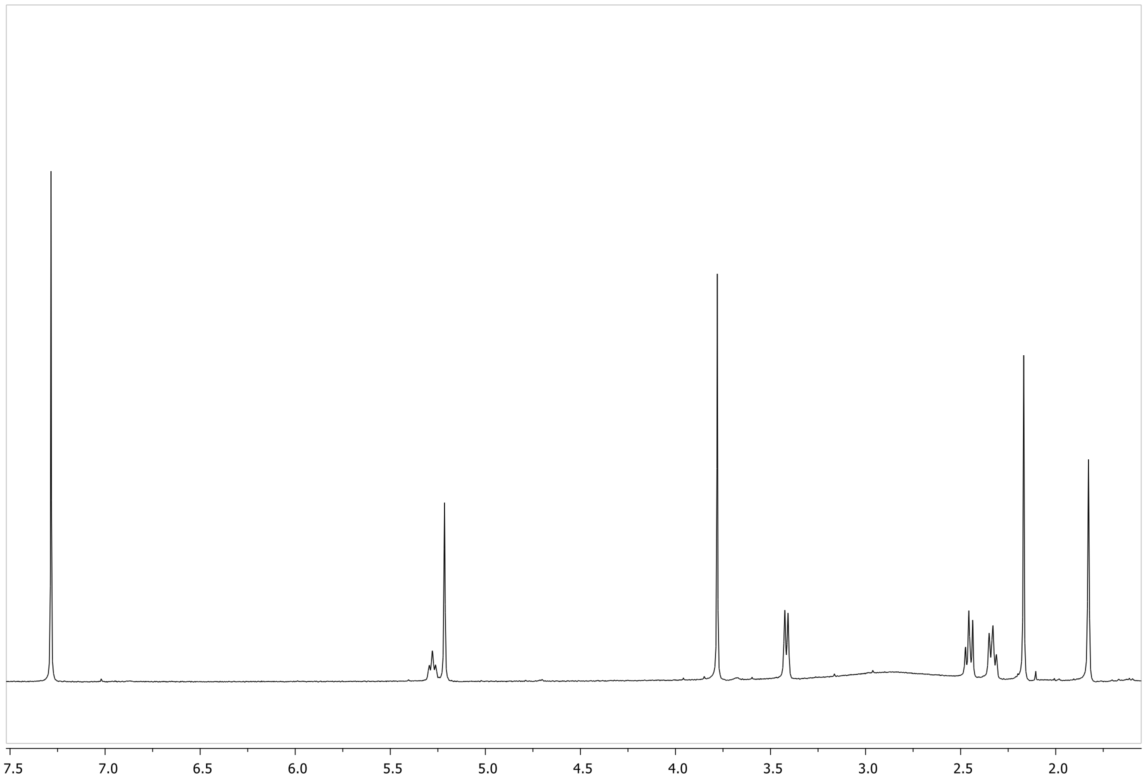

Figure S5. ^1H NMR spectrum of mycophenolic acid (**2**) recorded in $CDCl_3$ at 400 MHz.

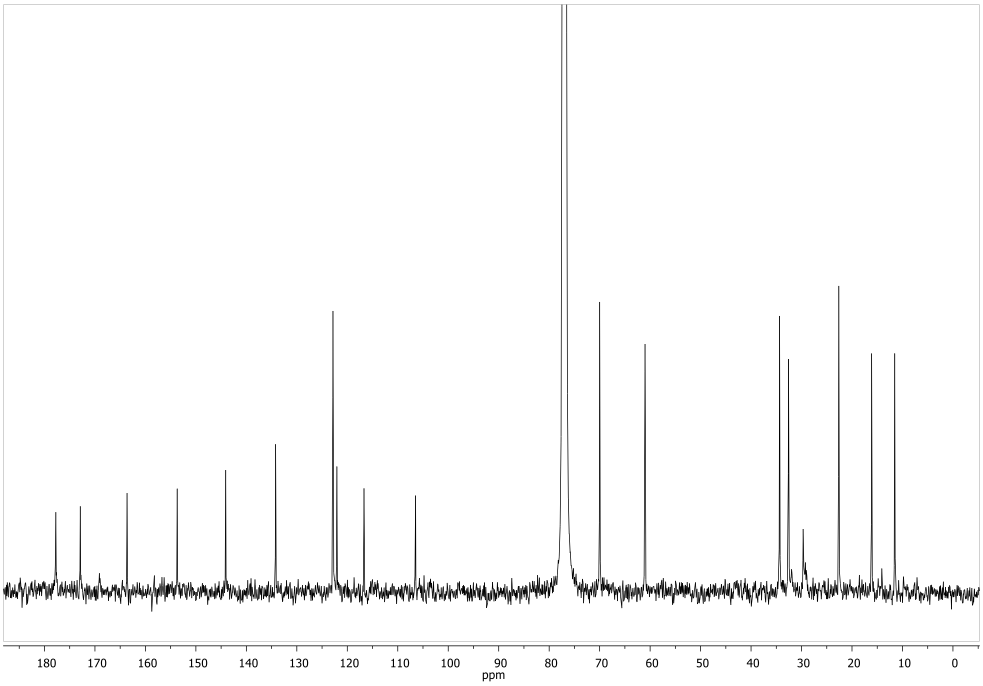

Figure S6. ^{13}C NMR spectrum of mycophenolic acid (**2**) recorded in $CDCl_3$ at 100 MHz.

Cis-bis(methylthio)silvatin (**1**)

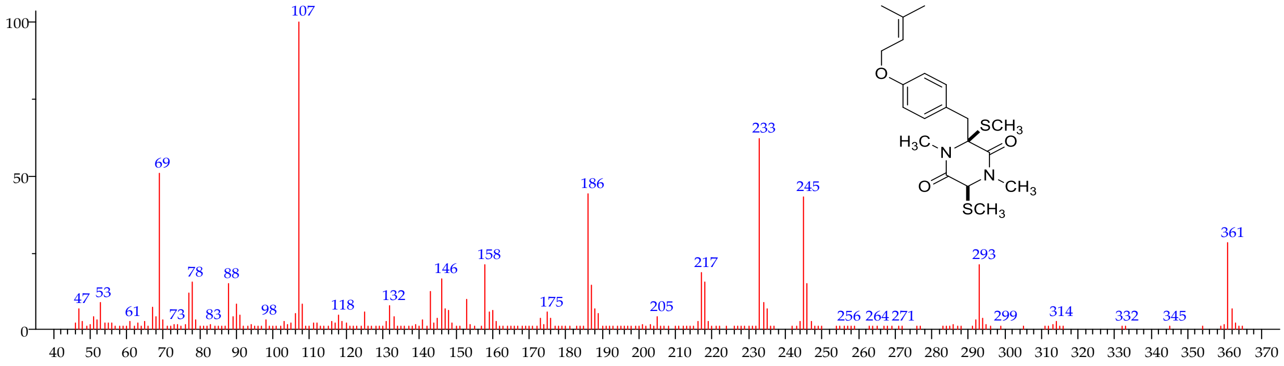

Saroclazine A/B (**2**)

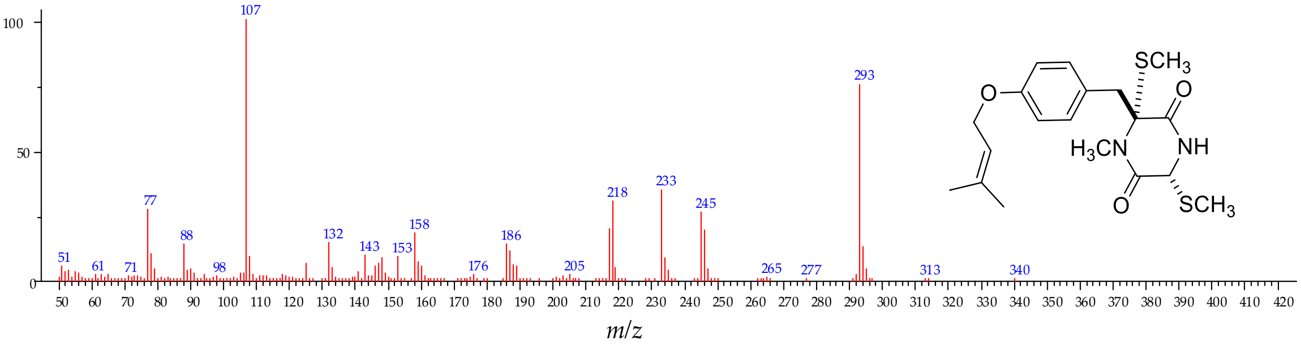

Fusaperazine E/F (**3**)

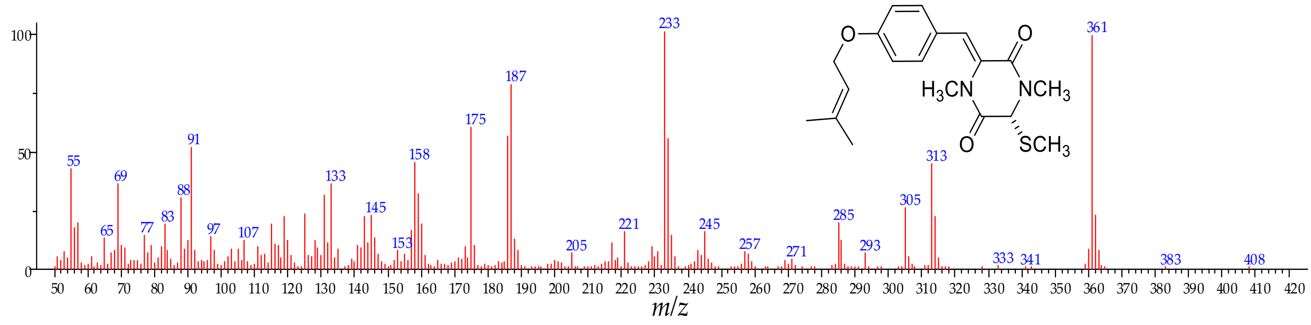

6-Oxomethylthiosilvatin (**4**)

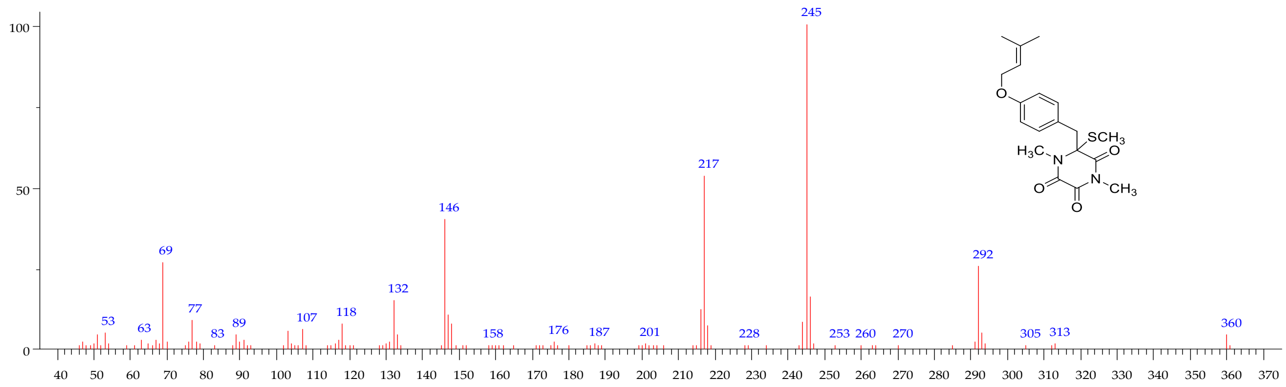

Bilain B (2TMS) (**5**)

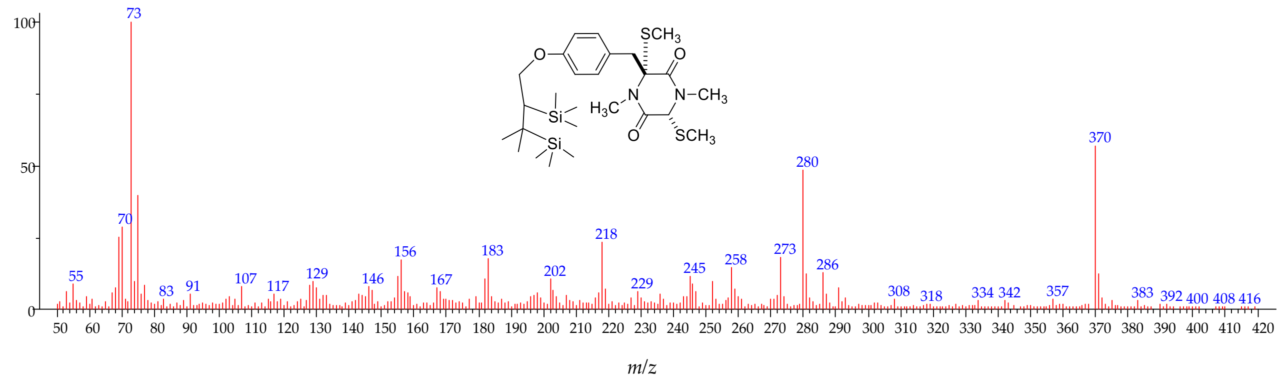

Deprenyl-bis(methylthio)silvatin (TMS) (**6**)

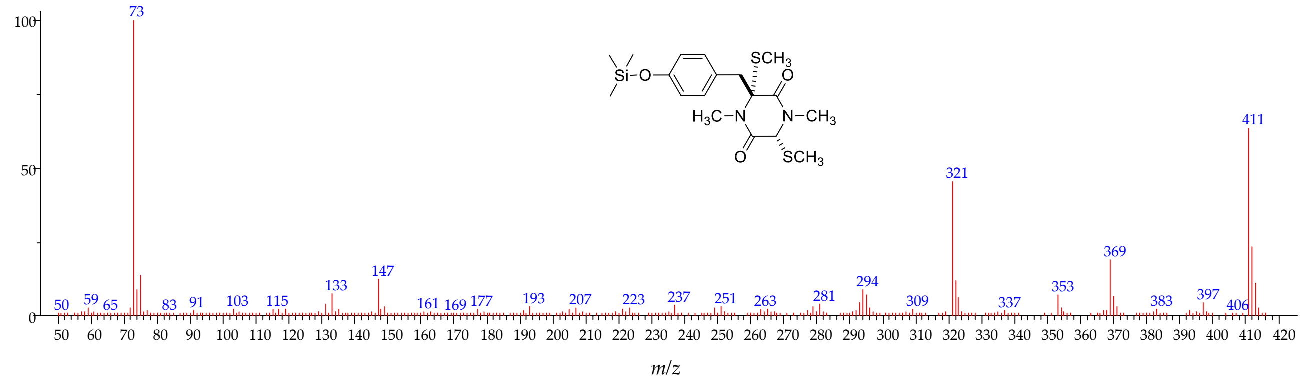

Fusaperazine A (TMS) (**7**)

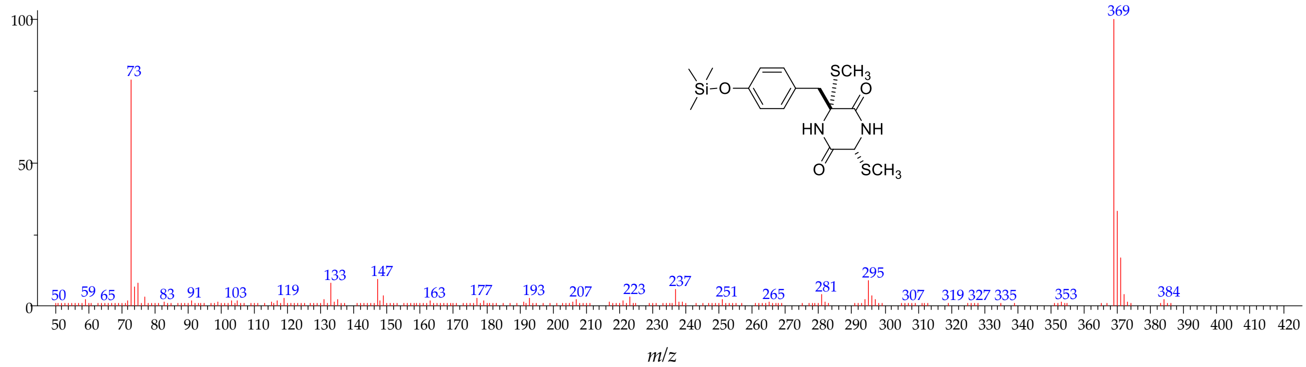

Mycophenolic acid (**8**)

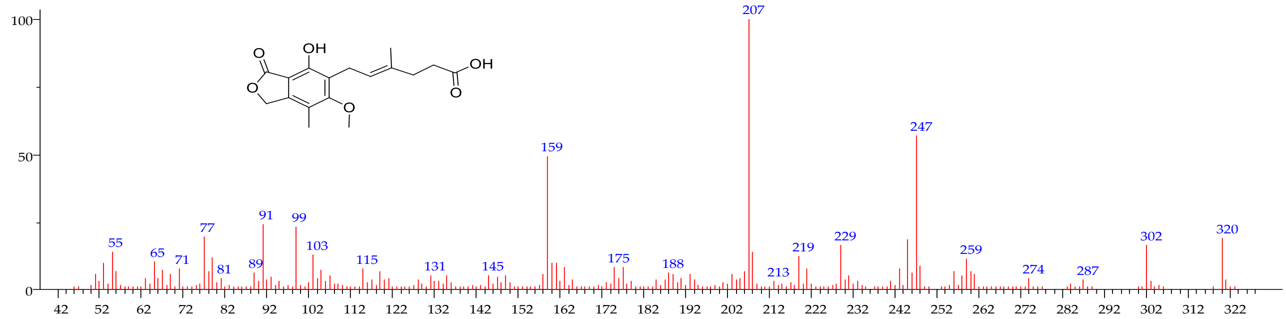

Brevianamide A/B (TMS) (**9**)

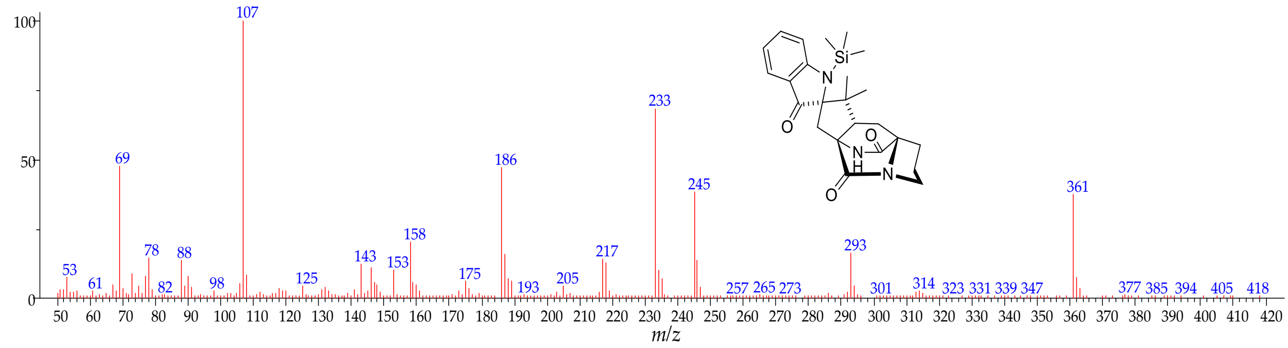

Figure S7. EI mass spectra at 70eV of identified metabolites.

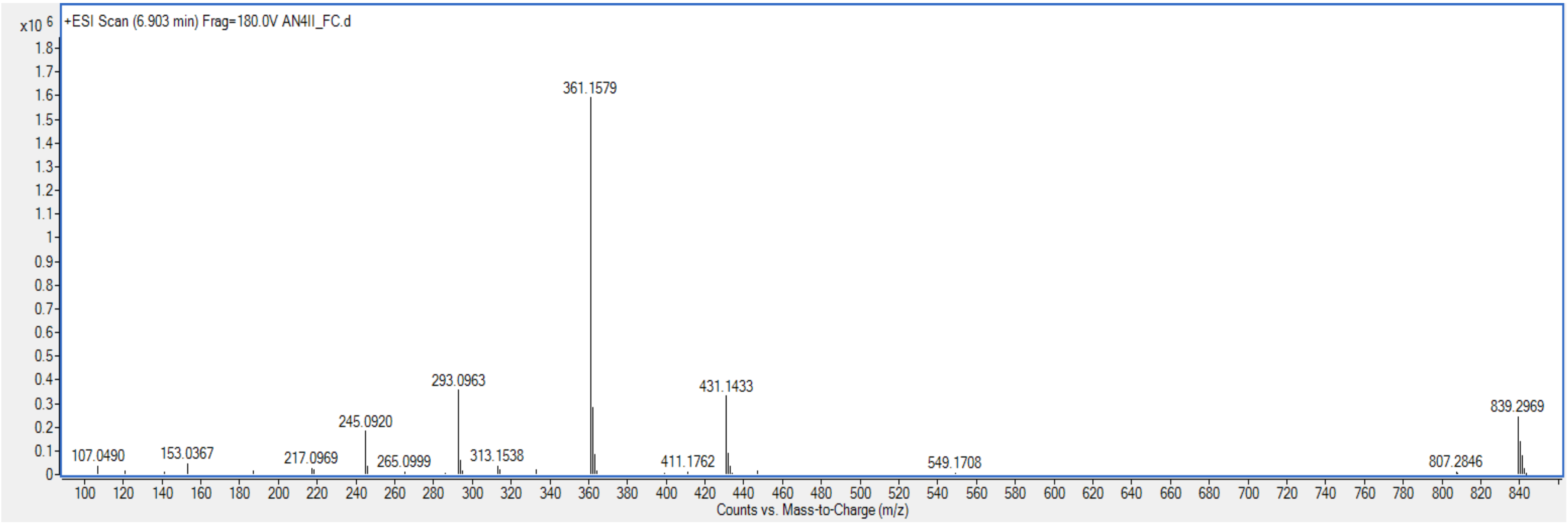

Figure S8. *Cis*-bis(methylthio)silvatin mass spectrum recorded in positive mode.

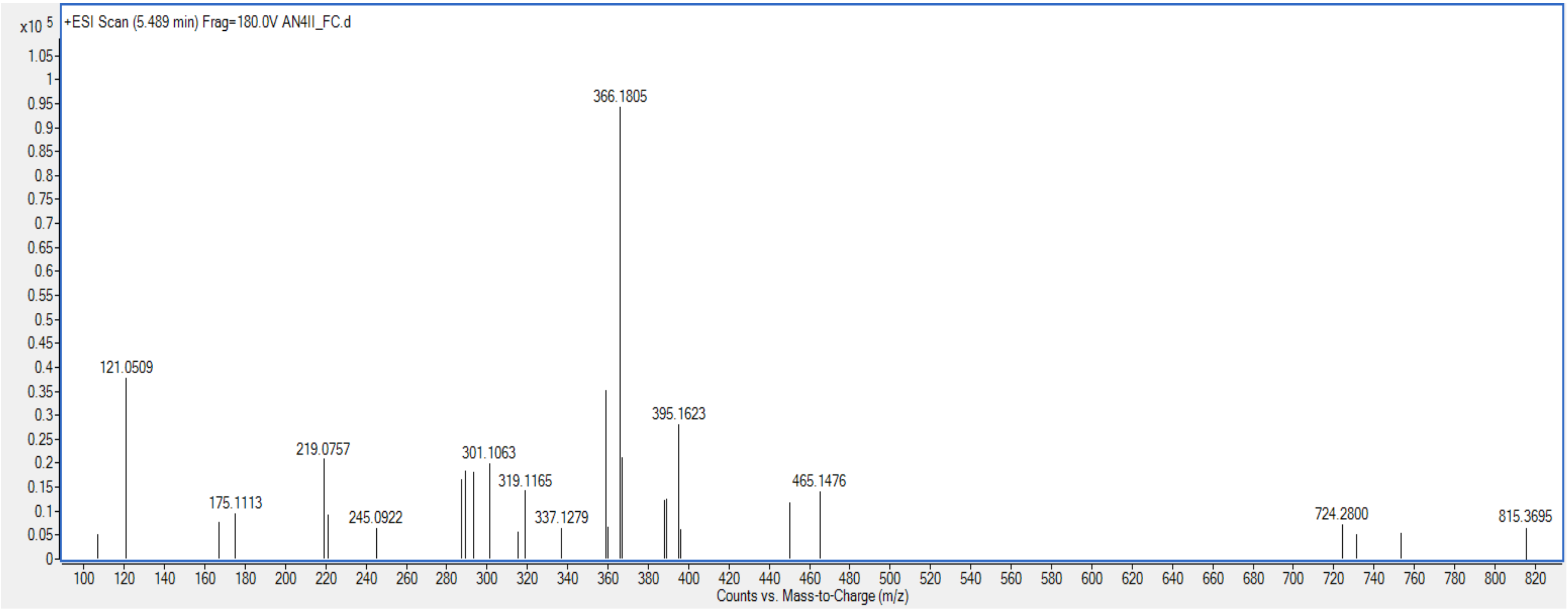

Figure S9. Saroclazine A/B mass spectrum recorded in positive mode.

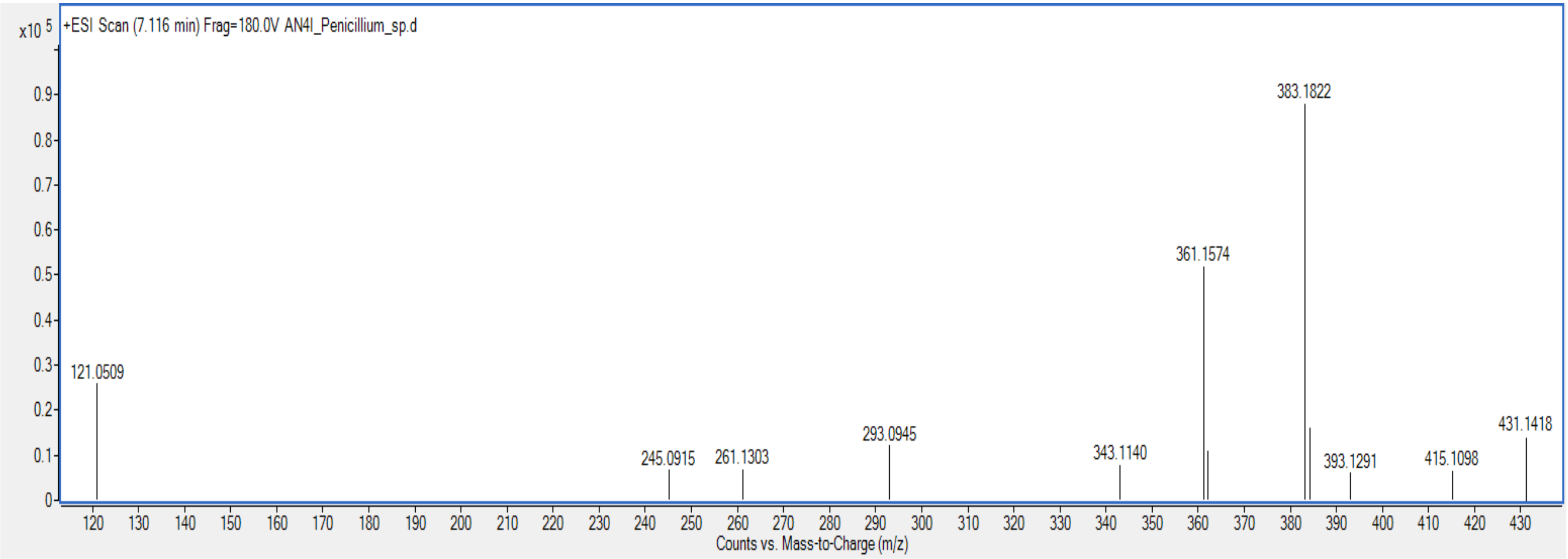

Figure S10. Fusaperazine E/F mass spectrum recorded in positive mode.

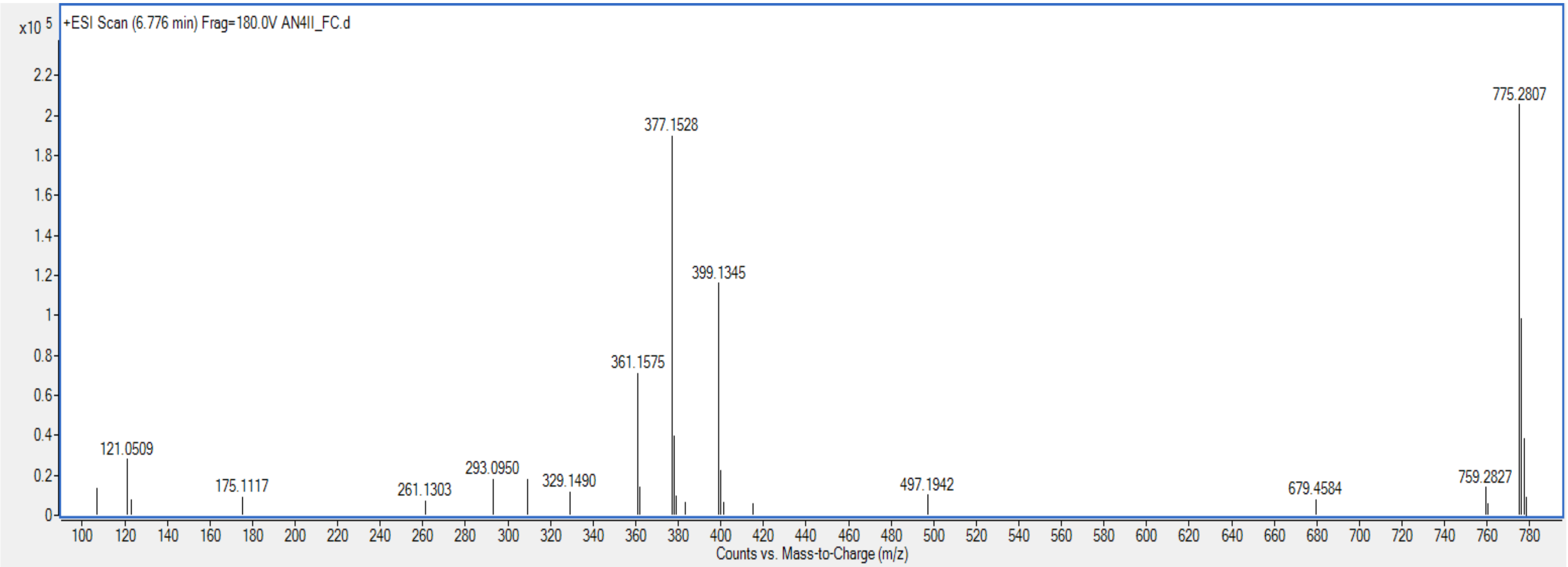

Figure S11. 6-Oxo-methylthiosilvatin mass spectrum recorded in positive mode.

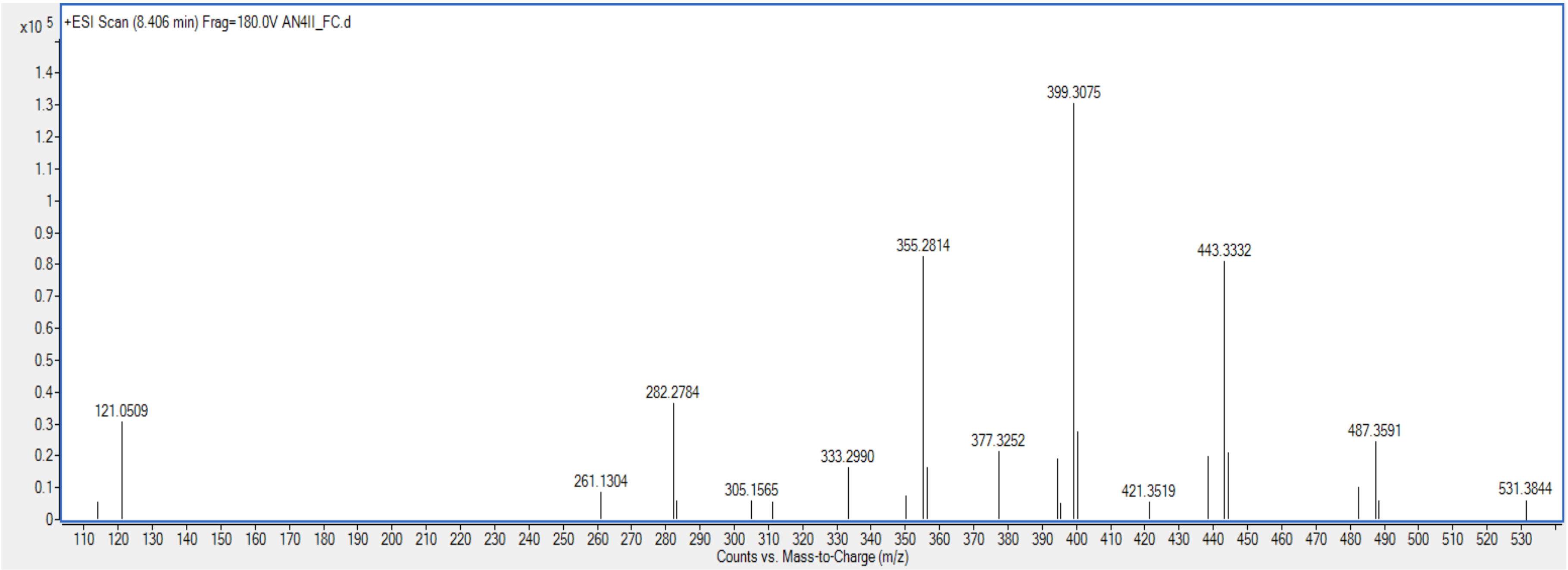

Figure S12. Bilain B mass spectrum recorded in positive mode.

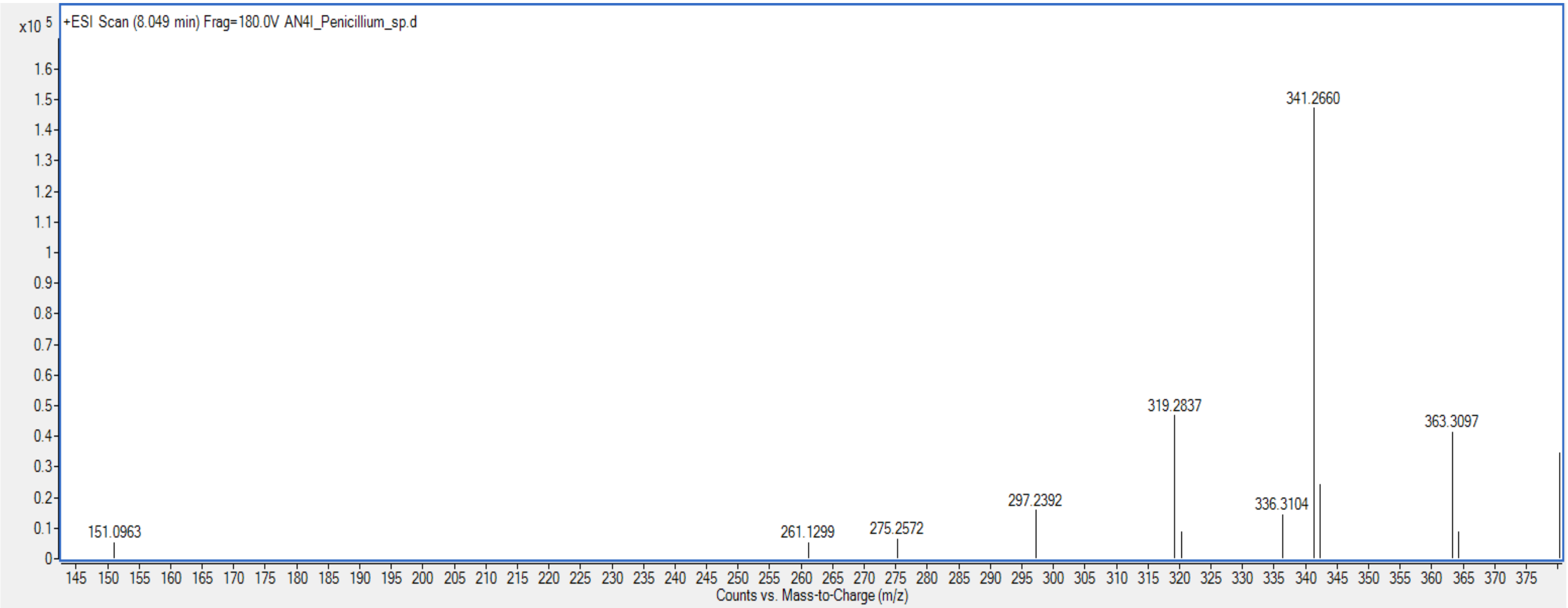

Figure S13. Deprenyl-bis(methylthio)silvatin mass spectra recorded in positive mode

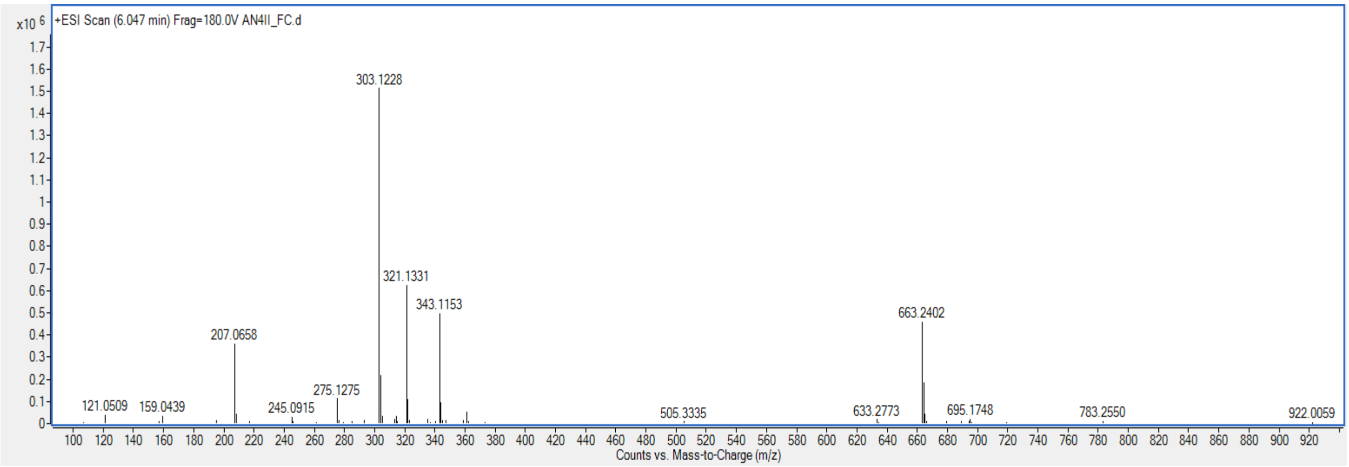

Figure S14. Mycophenolic acid mass spectrum recorded in positive mode.

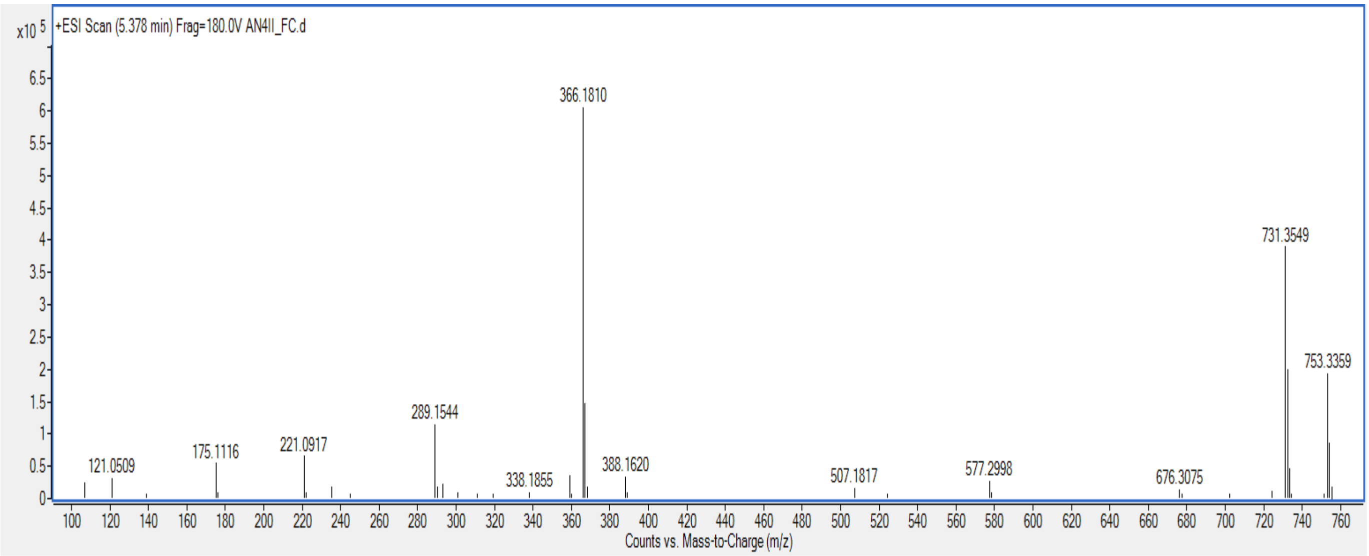

Figure S15. Brevianamide A/B mass spectrum recorded in positive mode.
